# Genomic insights into the diversity, virulence and resistance of *Klebsiella pneumoniae* extensively drug resistant clinical isolates

**DOI:** 10.1099/mgen.0.000613

**Published:** 2021-08-23

**Authors:** Amy H. Y Lee, William F. Porto, Célio de Faria Jr, Simoni C. Dias, Sérgio A. Alencar, Derek J. Pickard, Robert E. W. Hancock, Octavio L. Franco

**Affiliations:** ^1^​ Centre for Microbial Diseases and Immunity Research, University of British Columbia, 2259 Lower Mall Research Station, Vancouver, British Columbia, Canada; ^2^​ Department of Molecular Biology and Biochemistry, Simon Fraser University, South Sciences Building 7107, 8888 University Drive, Burnaby, British Columbia, Canada; ^3^​ S-Inova Biotech, Pós-graduação em Biotecnologia, Universidade Católica Dom Bosco, Campo Grande, MS, Brazil; ^4^​ Porto Reports, Brasília-DF, Brazil; ^5^​ Laboratório Central de Saúde Pública LACEN, Brasília, Brazil; ^6^​ Centro de Análises Proteômicas e Bioquímicas, Pós-graduação em Ciências Genômicas e Biotecnologia, Universidade Católica de Brasília, Brasília-DF, Brazil; ^7^​ Pós-graduação em Biologia Animal, Universidade de Brasília, Campus Universitário Darcy, Brasília, Brazil; ^8^​ Welcome Trust Sanger Institute, Hinxton, Cambridge, UK

**Keywords:** CC258, *Klebsiella pneumoniae*, β-lactamase, pan-genome, ST11, XDR

## Abstract

*
Klebsiella pneumoniae* has been implicated in wide-ranging nosocomial outbreaks, causing severe infections without effective treatments due to antibiotic resistance. Here, we performed genome sequencing of 70 extensively drug resistant clinical isolates, collected from Brasília’s hospitals (Brazil) between 2010 and 2014. The majority of strains (60 out of 70) belonged to a single clonal complex (CC), CC258, which has become distributed worldwide in the last two decades. Of these CC258 strains, 44 strains were classified as sequence type 11 (ST11) and fell into two distinct clades, but no ST258 strains were found. These 70 strains had a pan-genome size of 10 366 genes, with a core-genome size of ~4476 genes found in 95 % of isolates. Analysis of sequences revealed diverse mechanisms of resistance, including production of multidrug efflux pumps, enzymes with the same target function but with reduced or no affinity to the drug, and proteins that protected the drug target or inactivated the drug. β-Lactamase production provided the most notable mechanism associated with *K. pneumoniae *. Each strain presented two or three different β-lactamase enzymes, including class A (SHV, CTX-M and KPC), class B and class C AmpC enzymes, although no class D β-lactamase was identified. Strains carrying the NDM enzyme involved three different ST types, suggesting that there was no common genetic origin.

## Data Summary

Genomic data generated in this study are publicly available and can be accessed at the EMBL-EBI European Nucleotide Archive: accession number ERP010411.

**Impact Statement**

*
Klebsiella pneumoniae* is an important Gram-negative pathogen with high levels of antimicrobial resistance. Of particular concern is the emergence of extensively drug resistant (XDR) strains that are susceptible to at most only two antimicrobial classes; thus, limiting potential treatments. In this study, we performed comparative genomic analyses of 70 XDR clinical isolates that were collected from Brasília’s hospitals (Brazil) between 2010 and 2014. This collection is largely predominated by clonal complex 258 (CC258) with sequence types (STs) ST11 and ST340, but no ST258 isolates were identified. Furthermore, our analysis identified diverse resistance mechanisms that explain the extensive drug resistant phenotypes, with more than half of the isolates expressing extended-spectrum β-lactamases, all but one isolate expressing a carbapenemase, and five isolates having both a carbapenemase and colistin resistance.

## Introduction

*Klebsiella pneumoniae* is a Gram-negative bacterium that has been implicated in extensive outbreaks of nosocomial infections, ranging from pneumonia to urinary tract infections, meningitis, sepsis and abscesses [1–4]. *K. pneumoniae* is considered by the World Health Organization as a ‘priority pathogen’ due to its high levels of antimicrobial resistance [5], including the increasingly common emergence of extensively drug resistant (XDR) strains with very few therapeutic options. These XDR *K. pneumoniae* strains (i.e. those that are susceptible to at most only two antimicrobial classes) have been reported globally, especially in hospital settings [6–9]. Overall, many antibiotic-resistance mechanisms can be identified in *K. pneumoniae *, with resistance to β-lactams having the greatest impact on treatment. Strains demonstrate intrinsic resistance to β-lactams mediated through the SHV β-lactamase encoded on the chromosome. In addition, plasmid-mediated β-lactamases, such TEM-1 and AmpC, as well as extended-spectrum β-lactamases (ESBLs) further contribute to its resistance repertoire [10]. Due to a lack of effective antimicrobial agents and underlying comorbidities of patients, ESBL-producing and carbapenem-resistant *K. pneumoniae* infections often reach mortality rates ranging from 23 to 75 %, representing far higher rates of morbidity and mortality than infections with non-resistant bacteria [11, 12].

Over the last two decades, a group of hypervirulent *K. pneumoniae* isolates with hypermucoviscosity has emerged to cause highly invasive infections. These hypervirulent *K. pneumoniae* strains are capable of infecting healthy individuals, causing pyogenic liver abscess, bacteraemia, lung, neck and kidney abscesses, pneumonia, cellulitis, necrotizing fasciitis, myositis, meningitis, and endophthalmitis [5]. Recent evolutionary genomic analyses of >2200 *K *. *pneumoniae* genomes from around the world indicated that multidrug resistant (MDR) lineages are highly diverse, with high levels of chromosomal recombination, distinct surface polysaccharide loci and a variety of plasmids. In contrast, hypervirulent clones exhibit rare chromosomal recombination and limited plasmid diversity, indicating reduced potential for horizontal gene transfer [13]. This suggests that MDR lineages, and particularly XDR strains, pose a huge threat since they have greater potential to acquire virulence genes than the hypervirulent clones have to acquire antibiotic resistance.

Here, we report the genome sequences of 70 XDR *K. pneumoniae* isolates collected from Brasília’s hospitals (Brazil) between 2010 and 2014 to characterize their resistance mechanisms and genomic diversity. We performed pan-genome analysis and compared these Brazilian isolates with isolates from around the world to identify their phylogenetic lineages. We found that our collection is largely predominated by clonal complex 258 (CC258) and sequence types (STs) ST11 and ST340.

## Methods

### *K. pneumoniae* strains

Ninety-five *K. pneumoniae* clinical isolates resistant to carbapenem were retrieved from 12 hospitals from Brasília, Brazil, during the period of 2010 to 2014, and stored at Brasília’s Laboratório Central (LACEN-DF) at −80 °C (Table S1, available with the online version of this article). Species identification and antibiotic-resistance profiling were determined using the Vitek 2 system (bioMérieux); sequencing subsequently identified one isolate as *Klebsiella quasipneumoniae *.

### Growth conditions and DNA isolation

*K. pneumoniae* isolates were streaked onto MacConkey agar plates (Sigma-Aldrich), and sub-cultured into liquid Luria–Bertani (LB) medium (Sigma-Aldrich) for overnight growth at 37 °C. The DNA was isolated using the cationic detergent cetyltrimethylammonium bromide (CTAB) protocol, as described by Petersen and Scheie [14], with the minor modifications specified below. Bacterial cells from 2 ml of overnight culture were collected by centrifugation, and resuspended in 300 µl 2× CTAB lysis buffer (100 mM Tris-HCl pH 8.0, 1.4 M NaCl, 20 mM EDTA, 2 % CTAB and 0.2 % β-mercaptoethanol). The tube containing the suspension was then placed in a 65 °C water bath for 30 min to lyse the cells. Following incubation, one volume of chloroform:isoamylalcohol (24 : 1) was added to each sample. The samples were centrifuged and the aqueous phase was transferred to a new centrifuge tube. The genomic DNA in the aqueous phase was precipitated with ~400 µl cold isopropanol overnight, washed with 1 ml 70 % ethanol, and then resuspended in 100 µl Milli-Q water (Millipore). DNA concentration was assessed by fluorimetry using a Qubit system (Thermo-Fisher Scientific). DNA purity was assessed using a Thermo-Fisher Scientific NanoDrop 2000c spectrophotometer.

### Genome sequencing and assembly

Genome sequencing was performed according to Holt *et al.* [15]. Purified genomic DNA was sequenced via Illumina HiSeq 2000 at the Wellcome Trust Sanger Institute. Index-tagged paired-end Illumina sequencing libraries were prepared, combined into pools of uniquely tagged libraries and sequenced on the Illumina HiSeq 2000 to generate tagged 125 bp paired-end reads. Only isolates (*n*=79) with sequencing depth greater than 40× coverage were *de novo* assembled (Table S2). Sequencing quality of raw Illumina sequencing reads was assessed using FastQC v0.11.8 (https://www.bioinformatics.babraham.ac.uk/projects/fastqc/), followed by trimming with trimmomatic v0.39 [16] using the default parameters to remove Illumina adapters, low-quality bases and any reads less than 36 bp. Trimmed paired-end reads that overlapped (due to the original DNA fragments being shorter than twice the length of reads) were merged to generate longer single-end reads using FLASh v1.2.11 [17]. The remaining paired-end trimmed reads and the merged reads were subsequently used in *de novo* assembly with SPAdes v3.13.2 [18], followed by removal of contigs less than 200 bp with SeqKit v0.11.0 [19]. Genome assemblies were further assessed using quast v5.0.2 [20]. However, a subset of isolates with assembled genomes (*n*=5) did not include any associated sample metadata information (such as LACEN ID, sampling time and sample isolation sites) and were removed for all subsequent analyses. Isolates excluded from this analysis and the rationale for exclusion have been listed in Table S3. Raw sequencing reads have been deposited in the European Nucleotide Archive under accession number ERP010411.

### Genome annotation

All assembled genomes were annotated with the prokaryotic genome annotation pipeline, Prokka, v1.12 [21].

### Identification and classification of multilocus sequence type (MLST), virulence genes and resistance genes

To identify MLST, virulence genes and resistance genes from each isolate, we took two different approaches: (a) using raw sequencing reads with srst2 [22], a rapid molecular typing tool; and (b) analysing assembled genomes with Kleborate v0.4.0-beta (https://github.com/katholt/Kleborate), a tool for screening *Klebsiella* genome assemblies for important features [23–25]. The MLST marker genes for analysis were retrieved from the *Klebsiella* Pasteur MLST sequence definition database (https://bigsdb.pasteur.fr/klebsiella/). The rationale behind using multiple methods was to generate a robust set of MLSTs, virulence genes and resistance genes, especially given that the quality of genome assemblies would impact on allele identification, given the varied sequencing depth of a number of the isolates. The K and O locus types were subsequently detected from assemblies using Kleborate v0.4.0-beta [26].

To understand the genomic context of the carbapenemase (*bla_KPC_*) gene and its associated replicative transposon *Tn*4401, we used TETyper [27] to track sequence variation within *Tn*4401 to infer transposition events and potential *bla_KPC_* transmission. We used the *Tn*4401b sequence from *pKPC*_UVA01 (CP017937) as our reference sequence, and provided both assembled genomes as well as raw fastq reads to identify transposon structural variation, as well as the genomic context of *Tn*4401b.

### Pan-genome and phylogenetic analyses

To perform pan-genome and gene presence–absence analyses, we further excluded two assemblies with >800 contigs (2 714 082 and 3 919 904) as contig breaks can impact gene presence–absence. An additional assembly (4213265) was removed due to poor species match in Kleborate and a larger than expected genome size (10.7 Mb). Thus, we performed pan-genome analyses for a final 70 assembled and annotated isolates with associated metadata (i.e. sample isolation sites, year or antibiotic-resistance profiles) using the rapid large-scale prokaryote pan-genome analysis pipeline Roary [28]. We also included 20 randomly selected reference genomes from diverse clonal groups of MDR, hypervirulent and unassigned lineages (Table S4) [13] to understand the distribution of these strains within the global context. Using GFF3 files from our Prokka-annotated *de novo* assemblies, and the National Center for Biotechnology Information (NCBI) reference genomes, a core-genome alignment was generated using Roary. Core-genome SNPs were extracted from this core-genome alignment using the SNP Sites program (https://github.com/sanger-pathogens/snp_sites) [29], and used as input to generate a maximum-likelihood tree with RAxML [30]. To create the best-scoring tree with bootstrapping support, we used the GTRCAT model with the –f a and the –N autoMRE options in RAxML. A gene presence–absence matrix was mapped onto the phylogenetic tree using the roary_plot function. The phylogenetic tree with associated *K*, *O* and antimicrobial-resistance genes was visualized using ggtree (https://bioconductor.org/packages/devel/bioc/vignettes/ggtree/inst/doc/ggtree.html).

### Pan-genome and phylogenetic analyses of ST11 isolates

Given that the majority of our isolates belongd to ST11, we performed additional analyses on ST11 isolates by comparing Brasília ST11 isolates to all publicly available ST11 reference-quality genomes. We downloaded 677 reference-quality *K. pneumoniae* genomes from the National Center for Biotechnology Information (NCBI) (Table S5). To identify reference-quality isolates that belonged to the ST11 group rapidly, we used *mlst* v2.19.0 (https://github.com/tseemann/mlst) and data from PubMLST [31] to identify 128 ST11 reference genomes (Table S5). The associated country of isolation (‘geo_loc_name’) was pulled from each associated BioProject using Entrez-direct v13.9 [32] as this geographical data was more complete than latitude and longitude (‘lat_lon’) data. Pan-genome analyses of Prokka-annotated Braslia ST11 and reference ST11 genomes were performed using Roary as described above. Core-genome SNPs were extracted as above, followed by creating a best-scoring maximum-likelihood tree with RAxML and visualization using ggtree.

### SNP differences between the ST15 isolate and the PMK1 reference

Multi-FASTA alignment of the core-genome SNPs between the ST15 isolate (6175670) and PMK1 was extracted from the above and visualized in MView [33]. Direct SNP calling via snippy v4.6.0 (https://github.com/tseemann/snippy), using PMK1 as the reference genome, was also performed.

## Results

### Genome sequencing and assembly of *K. pneumoniae* clinical isolates

Genome sequencing of 95 clinical isolates was performed. One isolate (5121467) was classified as *K. quasipneumoniae* subsp *. quasipneumoniae* (Table S6), and thirteen isolates had insufficient sequencing depth (less than 40×) for *de novo* assemblies. Additional isolates with no associated metadata or with poor assemblies were removed from subsequent comparative genomic analyses (see Methods). The final collection of 70 assembled genomes (Table S2) had a median of 71 contigs (minimum of 43 contigs and maximum of 238 contigs) and a median N50 (i.e. the shortest contig length that covers 50 % of the genome) for contig lengths of ~270 746 bp (Table S7). The median genome size was ~5.68 Mbp, with a range of 5.28 – 6.41 Mbp. These strains were isolated from twelve hospitals, and predominately came from urine (15) or urinary catheters (12), blood (14), pulmonary fluids (14) and a variety of other sites (15) (Table S1, Fig. S1a).

### *K. pneumoniae* diversity and pan-genome analysis

**Fig. 1.**
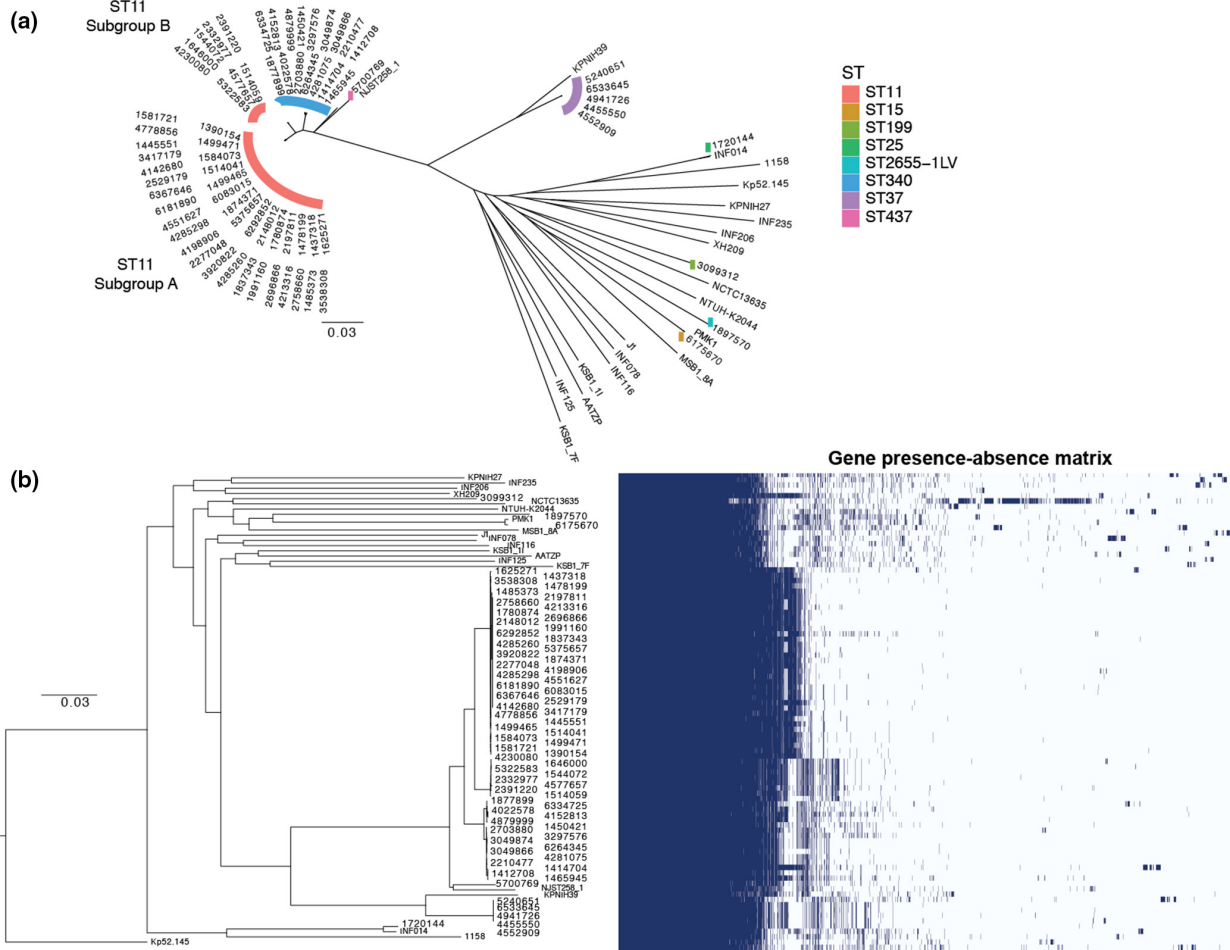
Core-genome SNP phylogeny and gene presence–absence matrix of 70 *K. pneumoniae* isolates from Brasília’s hospitals between 2010 and 2014, and 20 diverse *K. pneumoniae* reference genomes. Using the pan-genome analysis with Roary, we identified 4173 genes present in >95 % of strains (core genes), and an additional 14 316 genes present in the accessory genome. Core-genome SNPs were subsequently used to generate maximum-likelihood phylogenetic trees using RAxML. (**a)** Unrooted core-genome phylogeny with STs of the Brasília isolates highlighted in different colours. (**b)** The gene presence–absence matrix from the Roary pan-genome. Scale bar is a maximum likelihood estimate of the number of substitutions that have occurred on average per site between two nodes in a tree.

Genomic studies on global *K. pneumoniae* populations have illustrated their diversity, with hundreds of phylogenetic lineages or ‘clones’ [13, 15]. To gain further insights into these regional Brazilian isolates, we performed pan-genome analyses of these isolates together with 20 representative reference *K. pneumoniae* strains from diverse clonal groups to identify all genes found within this collection of strains (i.e. the pan-genome) [13] (Table S4). Specifically, we investigated the core-genome that contains genes shared by all strains, and the accessory genome that encodes genes found only in subsets of strains. Each genome assembly was annotated using Prokka, followed by Roary, to cluster orthologous coding sequences to create a matrix of gene presence or absence across the entire collection together with the 20 representative references genomes (Fig. 1). When we only considered the 70 Brasília isolates, we identified a core-genome of 4476 genes that were present in ≥95 % of isolates, with a large pan-genome of 10 366 genes (total number) (Table S8). When placed within the context of the larger *K. pneumoniae* species complex, by including the 20 reference genomes, we identified 4173 core genes present (~84 % of the total number of genes in each strain) in >95 % of strains. Furthermore, this identified an additional 14 316 genes present in the accessory genome (Table S8, Fig. S2). Core-genome SNPs were subsequently extracted from the Roary core-genome alignment and used to reconstruct a maximum-likelihood tree (Fig. 1) to reveal evolutionary relationships between strains. Overall, isolates from different hospitals and different time periods often grouped together in the phylogeny, and showed similar gene presence–absence ‘blocks’ (Figs 1b and S3). Thus, while overall there was no clear evidence of epidemic spread, we did observe persistence of certain lineages.

Typically, *K. pneumoniae* strains are classified according to their STs, a classification system based on nucleotide sequences at seven loci (*gapA*, *infB*, *mdh*, *pgi*, *phoE*, *rpoB* and *tonB*). Closely related STs with single locus variation are further assembled into CCs [34]. Within our collection, the majority (44/70) of isolates were identified as ST11, 16 isolates were ST340 and 5 isolates were ST37 (Figs 1a and S1b). However, despite its general predominance in Brasília’s hospitals, no ST258 isolate was identified in this XDR collection. In addition, one isolate each of ST15, ST25, ST199, ST2655-1LV and ST437 was identified (Table S6). The core-genome analyses supported the observation that the majority of isolates (61/70) belonged to a single CC, CC258 (comprising ST258, ST11, ST340, ST437 and ST512), with five isolates belonging to clonal group CC37 (comprising ST37) and others (Fig. 1).

The two predominant STs, ST11 and ST340, were found widely across different hospitals, with ST11 and ST340 isolated from nine and four hospitals, respectively (Fig. S1b). Furthermore, core-genome phylogeny indicated, in hospitals with multiple isolates from either ST11 or ST340 lineages, that these isolates from the same hospital tended to cluster together and could be sampled from different time points (Fig. S3). This potentially indicates that some of these *K. pneumoniae* isolates were persisting in the hospital during our sampling time period.

### Braslia ST11 isolates in relation to the global distribution of ST11

Given that the majority of strains within our collection belonged to the ST11 group, we were interested in understanding their phylogenetic relationships with ST11 isolates from around the world. In addition to the reference sequences included in Fig. 1, we included an additional 128 ST11 reference-quality genomes in order to reconstruct a detailed core-genome SNP phylogeny to visualize the geographical distribution of all ST11 strains from around the world. We observed that global ST11 isolates fell largely into many groupings, with the largest clade including most of the isolates from China (Fig. S4). This clade is distinct from the Braslia ST11 isolates, which were further separated into two distinct groups: ST11.A and ST11.B (Figs 1a and S4).

### *K. pneumoniae* virulence

There are four well-characterized *K. pneumoniae* virulence factors: capsule, lipopolysaccharide (LPS), fimbriae (type 1 and type 3) and siderophores [5]. The polysaccharide capsule is a key virulence factor, since isogenic non-capsulated strains cannot cause disease in murine infection models, when compared to capsulated strains [35]. The capsule also plays key defensive roles is suppressing the host inflammatory response, and reducing complement-mediated killing and phagocytosis. Traditionally, *Klebsiella* capsule types (K-types) are defined by serology, with 77 immunologically distinct K-types. In addition, nucleotide sequences of capsule synthesis loci (K loci) and molecular *K*-typing schemes have been used to provide finer resolution and discrimination than serological classification. Recent genomics studies [23] have further improved on these methods by establishing a curated K locus reference database with clear nomenclature and an efficient bioinformatics tool, Kaptive, to enable analysis [23, 26].

**Fig. 2.**
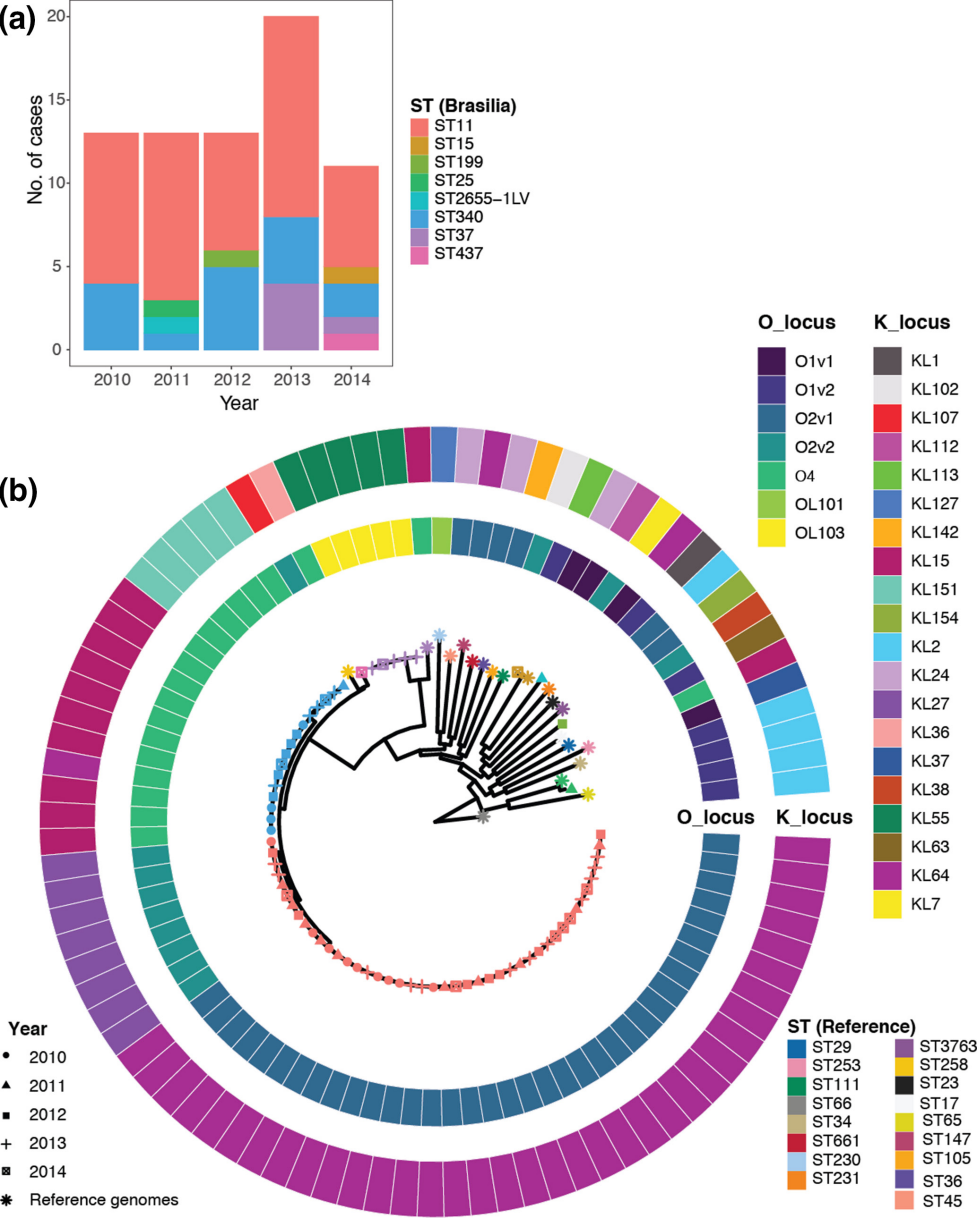
Distribution of ST, capsule biosynthesis (*K*) and LPS (*O*) loci. (**a**) Number of isolates of a particular ST sampled per year from Brasília’s hospitals, and (**b**) capsule and O-antigen serotype prediction mapped onto a circular core-genome SNP phylogeny, with tips labelled by sampling year (shape) and ST group (colour). Twenty reference-quality *K. pneumoniae* isolates (*) are included in the SNP phylogeny (**b**). For both (**a**) and (**b**), colour indicates ST groups, with Brasília isolates at the top and reference strains at the bottom.

We used Kaptive to identify K loci from our whole-genome sequences (Fig. 2). The predominant K loci included KL64 (37), KL15 (10) and KL27 (8). In addition, we also identified KL55 (5), KL151 (5), and one each of KL2, KL7, KL24, KL36 and KL154.

The other surface antigen of interest in *Klebsiella* is LPS, which includes lipid A, the core oligosaccharide and the O-antigenic polysaccharide. There are currently 12 distinct O loci identified, with both the O1 and O2 antigens the most common. In our collection, we identified a total of four different O loci including O2v1 (37 isolates), O2v2 (9), O4 (17), OL103 (5), and 1 each of O1v1 and O1v2 (Fig. 2). Surprisingly, while O2 was predominant, the O1 locus was only found in 2/70 strains.

**Fig. 3.**
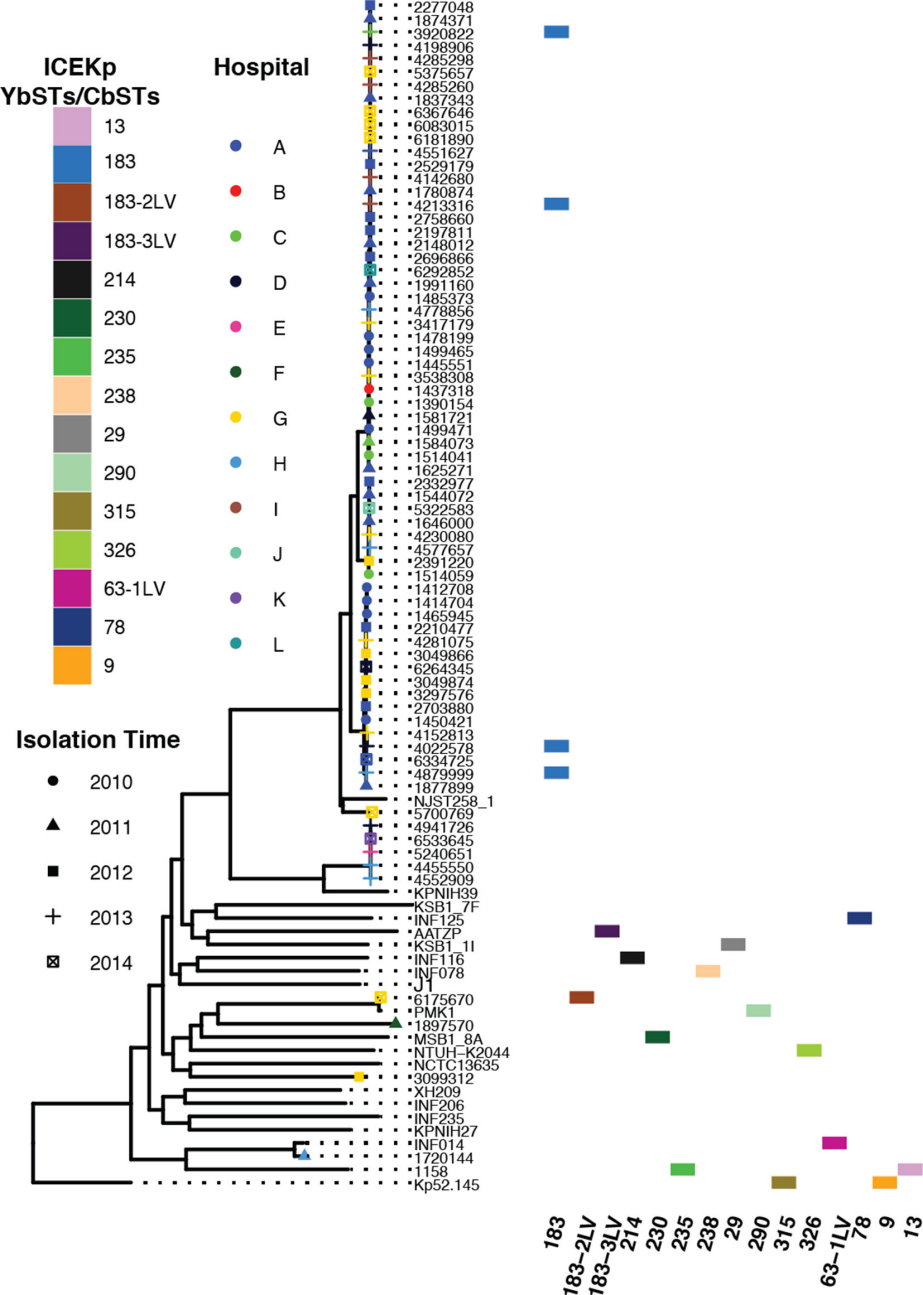
Presence of the yersiniabactin- and colibactin-encoding mobile element ICE*Kp* in a subset of Brasília *K. pneumoniae* isolates. The yersiniabactin STs (YbSTs) and colibactin STs (CbSTs) were identified by Kleborate. Core-genome SNP phylogeny of these isolates is shown on the left, with the tips of the tree labelled with the year of isolation (indicated by shapes) and hospital (indicated by colours). The majority of Brasília *K. pneumoniae* isolates do not have virulence genes.

Known *Klebsiella* virulence factors include the polyketide synthesis loci *ybt* and *clb*, which encode the iron-scavenging siderophore yersiniabactin and genotoxic colibactin, respectively. These two loci are located on the mobile genetic element ICE*Kp* [24]. Yersiniabactin plays a critical role in *Klebsiella* virulence since its iron-scavenging ability is not inhibited by human lipocalin-2, enabling it to promote bacterial growth and dissemination, while avoiding activating the host inflammatory response. Yersiniabactin is found in approximately a third of clinical isolates and is associated with strains isolated from bacteraemia and tissue-invasive infections. ICE*Kp* can carry additional genes, including those involved in the synthesis of the siderophore salmochelin (*iro*) and colibactin (*clb*). Given the emergent threat of MDR and XDR isolates becoming more virulent through the acquisition of virulence genes via acquisition of mobile genetic elements, we investigated the presence of virulence genes and the associated ICE*Kp* lineage in our collection. To look for the above virulence genes known to contribute to hypervirulence in *K. pneumoniae *, we used Kleborate, which assigns virulence STs (YbSTs for yersiniabactins and CbSTs for colibactins) and associated lineages using the locus-specific schemes of BIGSdb (https://bigsdb.pasteur.fr/klebsiella/klebsiella.html) [24]. By these criteria, only five of our clinical isolates carried ICE*Kp*, all of which were variants of ICE*Kp*3 with the yersiniabactin lineage *ybt9*, indicating potential increases in virulence in these isolates (Fig. 3). None of the isolates from this collection carried other virulence factors, such as colibactin, aerobactin (*iuc*), salmochelin (*iro*) or the *rmpA*/*rmpA2* genes associated with hypermucoidy. Interestingly, the five isolates that carried ICE*Kp3* were sampled from five different hospitals between 2013 and 2014, and isolated from either blood or pulmonary sites.

### Antibiotic-resistance profile

**Fig. 4.**
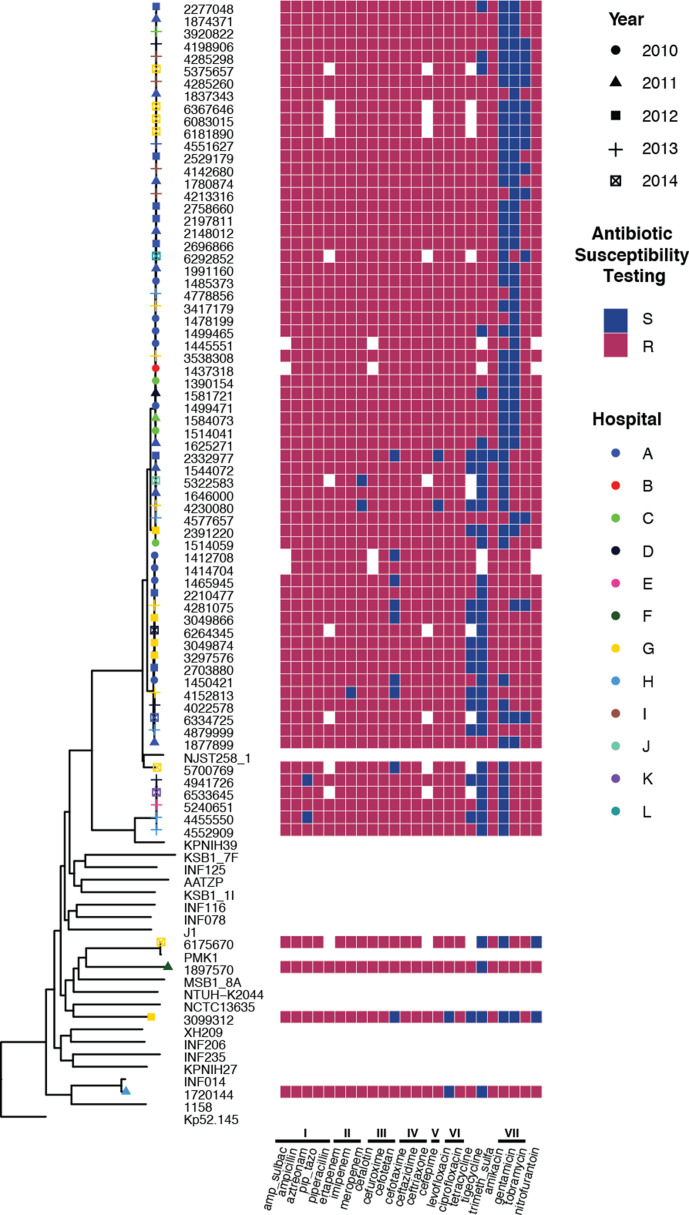
Heat map of the antibiotic-sensitivity profile of Brasília *K. pneumoniae* clinical isolates as assessed by Vitek 2. Resistance (red) or sensitivity (blue) against a particular antibiotic is defined using the European Committee on Antimicrobial Susceptibility Testing (EUCAST) clinical breakpoint at the time of isolation. Missing antibiotic information is shown in white. Antibiotics tested included antibiotics from the following classes (indicated by Roman numerals). (I) Penicillins, which included amp_sulbac (ampicillin/sulbactam), ampicillin, aztreonam, pip_tazo (piperacillin/tazobactam) and piperacillin. (II) Carbapenems, which included ertapenem, imipenem and meropenem. (III) Second-generation cephalosporins, which included cefalotin, cefuroxime and cefotetan. (IV) Third-generation cephalosporins, which included cefotaxime, ceftazidime and ceftriaxone. (V) Fourth-generation cephalosporins, which included cefepime. (VI) Quinolones, which included levofloxacin and ciprofloxacin. (VII) Aminoglycosides, which included amikacin, gentamicin, tobramycin. The complete profile with MIC values is available in Table S7.

Given our interest in understanding the XDR profiles of these isolates, we used the Vitek platform to characterize their antibiotic resistance (Fig. 4, Table S9). Almost all strains were resistant to β-lactam antibiotics (including penicillins, cephalosporins and carbapenems), quinolones, co-trimoxazole (a combination of trimethoprim and sulfamethoxazole), at least one aminoglycoside (although 57/70 showed susceptibility to at least one aminoglycoside) and nitrofurantoin. In contrast, 40 of the 70 clinical isolates were susceptible to the glycylcycline, tigecycline (see footnote in Table S9). Overall, the Brazilian clinical isolates demonstrated resistance to between 17 and 23 of the 24 tested antibiotics, and 8 to 11 of the 11 different tested antibiotic classes.

To understand the genetic basis for the high antimicrobial resistance observed in these isolates, we used the Kleborate program to identify both acquired resistance genes and chromosomal mutations known to be associated with drug resistance. Kleborate uses arg-annot [36] to classify antimicrobial-resistance genes, with the β-lactamases further delineated into Lahey classes. Kleborate assigns resistance scores from 0 to 3, with 0 as the least resistant (no ESBLs and no carbapenemases) and 3 being the most resistant (presence of carbapenemase with colistin resistance). Our collection contained highly resistant isolates where all except for one isolate expressed a carbapenemase (e.g. 64 isolates with a score of 2), with five isolates having both a carbapenemase and colistin resistance (score of 3) (Fig. S5).

**Fig. 5.**
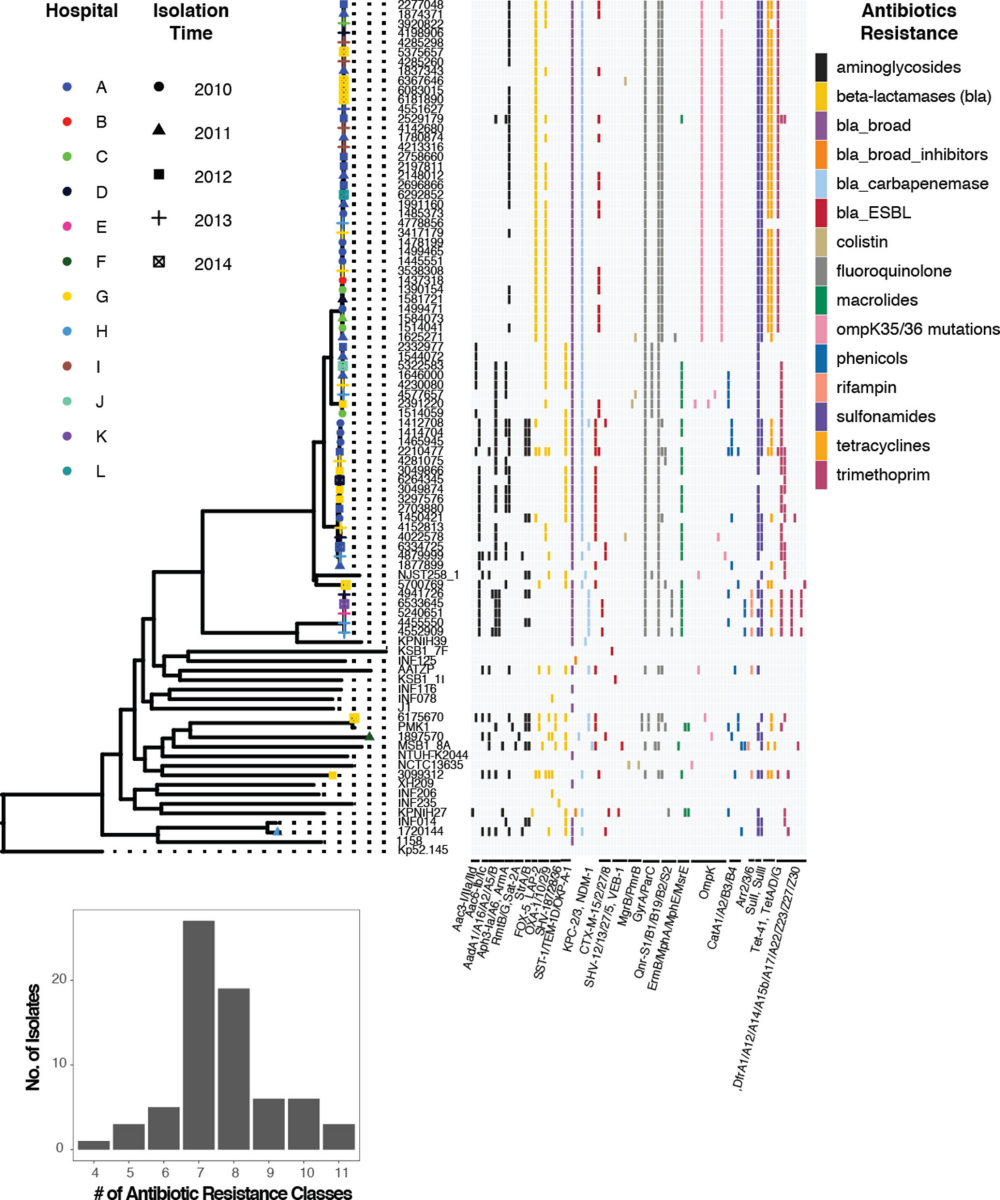
Antimicrobial resistance of *K. pneumoniae* clinical isolates from 12 Brasília hospitals. Brasília isolates sampled between 2010 and 2014 (with year of isolation shown by different shapes and hospital by different colours) mapped onto a core-genome SNP phylogeny reconstructed from 70 Brasília isolates and 20 diverse reference-quality *K. pneumoniae* strains. The presence of different resistance genes is coloured by class. The majority of the isolates have genes from at least seven or more antibiotic-resistance classes.

In *Klebsiella* species, colistin resistance is typically due to truncation or loss of the core chromosomal genes MgrB or PmrB. Both regulate LPS arabinosaminylation, which impacts polymyxin/colistin resistance, with MgrB being a negative-feedback regulator of the PhoP/PhoQ two-component regulatory system [37], while PmrB is the sensor kinase of another two-component regulatory system [38]. While none of the isolates in this collection had mutations in *pmrB*, five isolates from four different hospitals demonstrated either a complete loss or partial truncation of the *mgrB* gene (Fig. 5, Table S6); thus, these strains most likely would be resistant to colistin. Interestingly, the isolate (4022578) with the complete loss of the *mgrB* gene carried the virulence mobile genetic element ICE*Kp*3.

The tested antibiotics all have intracellular targets, and to traverse the outer membrane, antibiotics are filtered through the size-restricted water-filled channels of outer-membrane porins [39]. Loss of porins can mediate multidrug resistance. Half of the Brazilian clinical isolates had truncations in the major porin OmpK35 (40 %) in addition to a small channel variant of OmpK36, termed OmpK36GD [40] (total of 35/70 isolates; Table S6). In addition, three isolates had 95, 89 and 40 % truncations of only OmpK36, one had an *ompKTD* variant, two had OmpK35 truncations, and one had a deletion of K36. The absence of the *ompK36* gene and the *ompK36* mutation have been related to antibiotic resistance [40].

A second class of resistance mechanisms are represented by drug efflux, which is known to work in synergy with low outer-membrane permeability such as that due to porin deficiency. In the case of tetracycline, resistance was identified in 74 % of strains (43/58 tested). In *Klebsiella *, resistance is primarily mediated by efflux pumps encoded on mobile genetic elements. In the 70 clinical isolates studied here, two tetracycline efflux genes, *tetA* and *tetD*, were found in 37 isolates, while a further 10 isolates had only *tetD*, and 1 had only *tetA* for a cumulative carriage in 68 % of isolates.

Another set of resistances often carried on mobile genetic elements is aminoglycoside resistance mediated by modifying enzymes that add chemical constituents to the aminoglycosides and consequently inactivate them. Such enzymes were highly prevalent, being found in 91 % (64/70) of our clinical isolates. Indeed, resistance to amikacin (MIC ≥16 µg ml^−1^; 21/70), gentamicin (MIC ≥8 µg ml^−1^; 29/70) and tobramycin (MIC ≥8 µg ml^−1^; 55/70) was frequently observed. Similarly, for sulfamethoxazole/trimethoprim, *dfrA* promotes resistance to trimethoprim [41] and we found here that 96 % of isolates carried between one and three *dfrA* genes (often found on mobile genetic elements). For sulfamethoxazole, all except one isolate carried *sulI*, and 50 carried an additional *sulII* gene.

The fluoroquinolones act on topoisomerases in Gram-negative bacteria [42]. Point mutations in the binding domains termed the quinolone-resistance-determining regions (QRDRs) influence resistance. Here, we observed mutations in two topoisomerases, GyrA-83I and ParC80I, in most (69/70) isolates. In addition, we observed the plasmid-encoded *qnr* genes (primarily *qnrS1*) in 47/70 strains.

### β-Lactam resistance

The overuse and misuse of antibiotics has led to the rapid evolution of antibiotic-resistant *K. pneumoniae* strains. Overall, our XDR clinical isolates were nearly universally resistant to all tested classes of β-lactam antibiotics, including penicillins, carbapenems, and second-, third- and fourth-generation cephalosporins. There was substantial diversity in resistance mechanisms observed, but each strain presented two to nine different β-lactamase enzymes (Table S6, Fig. S5). The most widespread enzyme expressed was the broad spectrum β-lactamase SHV-11 (present in 67/70 isolates). Similarly, the majority of isolates expressed a class A β-lactamase KPC-2 (61/70) or a class B metallo-β-lactamase NDM-1 (7/70), while the class A ESBL GES-5 was found in one isolate. To understand the genomic context of *bla_KPC2_* and its associated replicative transposon *Tn*4401, we used TETyper and identified seven instances of truncations including ST11 strains 1625271, 4577657 and 6292852 (representing both ST11.A and ST11.B), and strains from four other ST groups including ST15 (4879999), ST25 (1720144), ST37 (4455550) and ST340 (3919904) [27].

NDM was first described in New Delhi, India, in 2009 [43], while the first reports of a NDM in Brazil were in 2013, in Rio Grande do Sul [44, 45], Rio de Janeiro and Brasília [46]. The NDM-producing strains identified in this study were in fact collected from 2013 to 2014, consistent with the previous reports. Of the seven NDM-producing strains (Table S6, Fig. S5), five, from four different hospitals, were all of the ST37 isolates identified and these formed a distinct phylogenetic group (Fig. 1b), suggesting they were highly related. The other two came from ST340, part of CC258, and a quite distinct and unique (in our collection) strain of ST15 (6175670). This isolate is ~600 core-genome SNPs and a total of 1359 SNPs different from the NDM-1-producing reference strain PMK1, the 2011 ‘source’ isolate from a Nepal hospital [47]. Consistent with our findings, NDM-producing strains from CC258 are rare, with a few case reports from Oman [48], New Zealand [49], England, Sweden and India [50].

Class A β-lactamases were also common, being found in 61/70 strains, with up to five enzymes found in two isolates. These include, in order of observation, LAP-2 (40 isolates) often found in conjunction with Oxa-2 (33), TEM1-D (19), and lower incidences of Oxa-9 (6), Oxa-1 (4), SHV-187 [3] and SHV-28 (1). ESBLs were identified in more than half (40/70 isolates), and included CTX-M−2 (18),–15 (15), −8 (5) and −27 (3).

## Discussion

MDR strains of the *Enterobacteriaciae* are isolates that are non-susceptible to at least one agent in ≥3 antimicrobial categories as defined by Margiorakos *et al.* [51] (see table 3 in their paper), while XDR isolates are non-susceptible to at least one agent in all but ≤2 categories. In particular, MDR *K. pneumoniae *, due to the spread of high-risk clones and widespread resistance plasmids, has become a major bacterial pathogen that causes hospital outbreaks worldwide with high morbidity and mortality [52, 53]. Some of these MDR isolates have evolved to become XDR isolates that have few therapeutic options [54]. By the above criteria, all of the isolates sequenced here are XDR, and arose from a single city during a 4 year period, enabling a detailed study of relationships between *K. pneumoniae* isolates that are highly recalcitrant to therapy. The isolates investigated here were resistant to 17–23/24 tested antibiotics, and 8–11/11 different tested antibiotic classes. Generally speaking, the most effective antibiotics were amikacin, gentamicin and/or tigecycline, but resistance to these antibiotics [based on European Committee on Antimicrobial Susceptibility Testing (EUCAST) definitions] were observed in 28.6, 41.4 and 42.8 % of isolates, respectively. This provides a very threatening picture given the continuous increase in resistance trends observed worldwide and evidence that some of these isolates form very closely related clusters suggesting clonal outbreaks.

Interestingly, there has been a worldwide spread of MDR ST258 *K. pneumoniae* that are limiting therapeutic options [52]. However, amongst our XDR isolates from Brasília’s hospitals, ST258 was absent and the major representative ST from CC258 was ST11. Nevertheless, with the continuous antibiotic-resistance development by bacteria, and the fact that there are strains already resistant to one or two last-choice antibiotics, it seems likely that no single therapeutic options will remain possible for all *K. pneumoniae* infections [52].

In these Brazilian hospital isolates, there were three STs, all from CC258, that made up the majority (87 %; 61/70) of strains. This included a single ST437 isolate, a cluster of 16 ST340 strains and a large number of ST11 isolates that appeared to form two sequence subgroups (termed ST11.A, comprising 36 strains, and ST11.B, comprising 8 isolates; Fig. 1a). The next largest class was the outgroup ST37, with five isolates, in addition to four other single isolates from ST15, ST25, ST199 and ST2655. These groups were more distantly related to the CC258 strains, but showed similarity with some reference strains. Thus, the 2014 ST15 isolate 6175670 was closely related to the 2011 Nepalese reference strain PMK1 (with a total of 1359 SNPs different between the two strains), but despite these strains bearing the carbapenemase NDM-1, they were quite distinct from the NDM-1-expressing ST37 cluster of five isolates. Similarly, the 2011 ST25 isolate 1720144 aligned closely with the most antibiotic-resistant hypervirulent reference strain INFO14 [13], presenting the worrying picture that hypervirulent isolates might be evolving to become untreatable.

One cluster of interest included ST340 and ST37 strains. ST340 strains were typified by KL15 (10 isolates), KL151 (5) or KL64 (1) capsule types, O4 LPS type, 1–4 AGMs (aminoglycoside modifying genes), common appearance of GyrA-83I and ParC-80I topoisomerase (fluoroquinolone-resistance) mutations, SulI (14/16 isolates), DfrA12 (14/16), few porin mutations (2/16), TEM-1D (10/16), and CTX-M ESBLs of various classes (15/16, most commonly CTX-M-15), as well as KPC-2 carbapenemase. In contrast, ST37 strains were capsule type KL55, O-type OL103, carried the three AGMs Aac3-IId^, AadA2^, AadA5, two fluoroquinolone-resistance loci, GyrA-83I, ParC-80I, all had DfrA12 plus other Dfr genes, lacked porin mutations and β-lactamases except carbapenemase NDM-1 and ESBL CTX-M-27 (3/5 strains). These results again are suggestive of highly related strains circulating in multiple hospitals in Brasília (each found in four hospitals), although the first isolation of ST37 strain was in 2013, suggesting this might be a more recently introduced lineage.

A key finding here was the existence of two very distinct clades of Brasília ST11 isolates as mentioned above. Further analysis showed these had quite distinct surface and antibiotic-resistance properties (Table 1). Thus, the 36 ST11.A isolates were all capsule type KL64 (39 % with missing genes) and LPS O-antigen type O2v1 (64 % with missing genes), while ST11.B isolates were KL27 and O2v2 (with only one missing O-antigen gene). Resistance also varied in a subtype-specific fashion. Thus, ST11.A generally had only the AGM AphA6 (25/36 isolates), with two having two additional AGMs. ST11.B lacked this enzyme and instead had between one and three of the enzymes Aac3-IIa, AadA2 and Aph3-Ia. Similarly with regards to fluoroquinolone resistance, while ST11.A strains had the mutations GyrA-83I, ParC-80I and Qnr-S1, ST11.B strains carried the former two mutations as well as GyrA-87G. Similar patterns were observed for other resistance genes: (i) ST11.A strains had 2–3 sulfonamide-resistance genes (*sulI*, *II*) with ST11.B strains only having *sulI*; (ii) 34/36 ST11.A strains carried the tetracycline efflux genes *tetAD* (one had only *tetD* and another none), while none of the ST11.B strains had either gene; (iii) 34/36 ST11.A strains had the trimethoprim dihydrofolate reductase gene *dfrA1*, while 6/8 ST11.B strains carried *dfrA12*; (iv) all ST11.A strains carried mutations in porins OmpK35 and 35/36 carried OmpK36 mutations, while only 2/8 ST11.B strains carried either; (v) with regards β-lactamases, ST11.A strains carried Lap-2 and 25/36 had Oxa-2; 15/36 had the ESBL CTX-M-2, while 6/8 ST11.B strains carried Oxa-2 and/or TEM-1D, 2/8 carried CTX-M-2, while both ST11.A and ST11.B carried KPC-2 and SHV-11. Thus, it is clear that these are two related but separate lineages. Intriguingly, both were first identified in 2010, and were identified annually until the last sampling period in 2014 in eight (ST11.A) and five hospitals (ST11.B) from multiple types of clinical samples, with both isolates isolated from four common hospitals. In particular, the predominance of KL64 ST11.A strains is of particular interest having been found as early as 2010, since in China this isolate arose much later (2016) [2]. It was curious that clone ST258, found in many Brazilian states, had never been identified in the large city of Brasília (from which these isolates came), despite extensive surveillance since 2010 [55, 56]. Nevertheless, the presence and persistence of a clone of ST11 in the capital, for at least a decade, provides considerable concern since this clone frequently presents as polymyxin resistant, removing one of the last resources against *Enterobacteriaciae* resistant to carbapenems [57].

**Table 1.** Characteristics of the sequenced strains

Strain*	ST	Year	Source	Hospital	K locus	O locus	No. of resistance genes/ classes	Resistance gene								
								AGM†	FQ‡	Sul§	Tet||	Trm/ Dfr¶	Omp mutants#	β- Lactamase**	Carbapene -mase	ESBL
1 390 154	ST11.A	2010	Urinary	C	KL64	O2v1	19/9	1	1, 2, 3	I, I*, II	AD	A1	K35, 36	L2, O2	KPC-2	CTX-M-2
1 437 318	ST11.A	2010	Pulm.	B	KL64	O2v1	21/10	–	1, 2, 3	I, I, II	AD	A1	K35, 36	L2, O2	KPC-2	CTX-M-2
1 445 551	ST11.A	2010	Urinary	A	KL64	O2v1††	22/10	–	1, 2, 3	I?, II	AD	A1	K35, 36	L2, O2	KPC-2	–
1 478 199	ST11.A	2010	Wound	A	KL64	O2v1	22/11	–	1, 2, 3	I, II	AD	A1	K35, 36	L2, O2	KPC-2	–
1 485 373	ST11.A	2010	Urinary	A	KL64	O2v1	21/10	–	1, 2, 3	I, I, II	AD	A1	K35, 36	L2, O2	KPC-2	CTX-M-2
1 499 465	ST11.A	2010	Urinary	A	KL64	O2v1††	22/11	–	1, 2, 3	I, II	AD	A1	K35, 36	L2, O2	KPC-2	–
1 499 471	ST11.A	2010	Tissue	A	KL64	O2v1	20/9	–	1, 2, 3	I, I, II	AD	A1	K35, 36	L2, O2	KPC-2	CTX-M-2
1 514 041	ST11.A	2010	Blood	C	KL64	O2v1††	21/10	1	1, 2, 3	I, I, II	AD	A1	K35, 36	L2, O2	KPC-2	CTX-M-2
1 581 721	ST11.A	2011	Pulm.	D	KL64	O2v1	21/10	1	1, 2, 3	I, II	AD	A1	K35, 36	L2, O2	KPC-2	–
1 584 073	ST11.A	2011	Blood	C	KL64	O2v1††	21/9	–	1, 2, 3	I, I, II	AD	A1	K35, 36	L2, O2	KPC-2	CTX-M-2
1 625 271	ST11.A	2011	Bone	A	KL64††	O2v1	22/10	–	1, 2, 3, 5	I, II	D	–	K35, 36	L2, O2	KPC-2	–
1 780 874	ST11.A	2011	Urinary	A	KL64	O2v1††	23/10	1	1, 2, 3	I, I, II	AD	A1	K35, 36	L2, O2	KPC-2	CTX-M-2
1 837 343	ST11.A	2011	Other	A	KL64	O2v1	22/11	–	1, 2, 3	I, II	AD	A1	K35, 36	L2, O2	KPC-2	CTX-M-2
1 874 371	ST11.A	2011	Blood	A	KL64	O2v1††	22/11	1	1, 2, 3	I, I, II	AD	A1	K35, 36	L2, O2	KPC-2	CTX-M-2
1 991 160	ST11.A	2011	Blood	A	KL64	O2v1	21/10	1	1, 2, 3	I, I, II	AD	A1	K35, 36	L2, O2	KPC-2	CTX-M-2
2 148 012	ST11.A	2011	Pulm.	A	KL64	O2v1††	18/8	1	1, 2, 3	I, I, II	AD	A1	K35, 36	L2, O2	KPC-2	CTX-M-2^
2 197 811	ST11.A	2012	Urinary	A	KL64	O2v1††	20/9	1	1, 2, 3	I, II	AD	A1	K35, 36	L2, O2	KPC-2	–
2 277 048	ST11.A	2012	Pulm.	A	KL64	O2v1††	22/9	1	1, 2, 3	I, I, II	AD	A1	K35, 36	L2, O2	KPC-2	CTX-M-2
2 529 179	ST11.A	2012	Other	A	KL64††	O2v1††	21/9	1, 3, 4	1, 2, 3	I, I, II	AD	A1+	K35, 36	L2, O2	KPC-2	CTX-M-2
2 696 866	ST11.A	2012	Urinary	A	KL64	O2v1	22/9	1	1, 2, 3	I, I, II	AD	A1	K35, 36	L2, O2	KPC-2	CTX-M-2
2 758 660	ST11.A	2012	Pulm.	A	KL64††	O2v1††	22/11	1	1, 2, 3	I, II	AD	A1	K35, 36	L2	KPC-2	–
3 417 179	ST11.A	2013	Pulm.	G	KL64	O2v1	19/9	1	1, 2, 3	I, II	AD	A1	K35, 36	L2, O2	KPC-2	–
3 538 308	ST11.A	2013	Urinary	G	KL64	O2v1	22/11	–	1, 2, 3	I, I, II	AD	A1	K35, 36	L2, O2	KPC-2	CTX-M-2
**3 920 822**	ST11.A	2013	Blood	C	KL64	O2v1††	19/9	1	1, 2, 3	I, II	A	A1	K35	L2, O2	KPC-2	–
4 142 680	ST11.A	2013	Blood		KL64††	O2v1††	22/11	1	1, 2, 3	I, II	AD	A1	K35, 36	L2	KPC-2	–
4 198 906	ST11.A	2013	Tissue	D	KL64††	O2v1††	21/10	1	1, 2, 3	I, II	AD	A1	K35, 36	L2	KPC-2	–
**4 213 316**	ST11.A	2013	Blood		KL64††	O2v1††	22/10	1	1, 2, 3	I, II	AD	A1	K35, 36	L2	KPC-2	–
4 285 260	ST11.A	2013	Blood		KL64††	O2v1††	20/9	1	1, 2, 3	I, II	AD	A1	K35, 36	L2	KPC-2	–
4 285 298	ST11.A	2013	Urinary		KL64††	O2v1††	20/9	1	1, 2, 3	I, II	AD	A1	K35, 36	L2	KPC-2*	–
4 551 627	ST11.A	2013	Other	A	KL64††	O2v1††	22/11	1	1, 2, 3	I, II	AD	A1	K35, 36	L2	KPC-2	–
4 778 856	ST11.A	2013	Urinary	H	KL64	O2v1	22/11	–	1, 2, 3	I, II	–	–	K35, 36	L2, O2	KPC-2	–
5 375 657	ST11.A	2014	Urinary	G	KL64††	O2v1††	22/10	1	1, 2, 3	I, II	AD	A1	K35, 36	L2	KPC-2	–
6 083 015	ST11.A	2014	Wound	G	KL64††	O2v1††	20/9	1	1, 2, 3	I, II	AD	A1	K35, 36	L2	KPC-2	–
6 181 890	ST11.A	2014	Urinary	G	KL64††	O2v1††	17/8	1	1, 2, 3	I, II	AD	A1*	K35, 36	L2	KPC-2	–
6 292 852	ST11.A	2014	Urinary	L	KL64††	O2v1††	22/22	1	1, 2, 3	I, II	AD	A1	K35, 36	L2	KPC-2	–
6 367 646	ST11.A	2014	Urinary	G	KL64††	O2v1††	18/9	–	1, 2, 3	I, II	AD	A1	K35, 36	L2	KPC-2	–
1 514 059	ST11.B	2010	Blood	C	KL27	O2v2	21/10	2, 3	1, 2, 4		–	A12	–	T1D	KPC-2	CTX-M-2
1 544 072	ST11.B	2011	Urinary	A	KL27	O2v2††	22/11	2	1, 2, 4		–	–	–	O2, T1D	KPC-2	–
1 646 000	ST11.B	2011	Urinary	A	KL27	O2v2	22/11	2, 3, 4	1, 2, 4		–	A12	–	O2, T1D	KPC-2	–
2 332 977	ST11.B	2012	Pulm.	A	KL27	O2v2	22/11	2	1, 2, 4		–	–	–	O2, T1D	KPC-2	–
2 391 220	ST11.B	2012	Other	G	KL27	O2v2	17/8	3	1, 2, 4		–	A12	K35, 36	O2	KPC-2	CTX-M-2
4 230 080	ST11.B	2013	Pulm.	G	KL27	O2v2	21/9	2, 3, 4	1, 2, 4		–	A12	–	O2, T1D	KPC-2	–
4 577 657	ST11.B	2013	Urinary	H	KL27	O2v2	21/10	3, 4	1, 2, 4		–	A12	K36	–	KPC-2	–
5 322 583	ST11.B	2014	Urinary	J	KL27	O2v2	17/8	2, 3, 4	1, 2, 4		–	A12	–	O2, T1D	KPC-2	–
**6 175 670**	ST15	2014	Pulm.	G	KL24††	O1v1	18/8	1, 2, 3	1, 2, 3, 4	II	D	A14	K35	T1D, O^+^;S	NDM-1	CTX-M-15
3 099 312	ST199	2012	Blood	G	KL154††	O2v1	22/9	1, 2, 3	1, 2, 3	I, I, II	AD	A1+	–	L2, O2, O^+^, S	KPC-2	CTX-M-2
1 720 144	ST25	2011	Urinary	H	KL2	O1v2	22/11	1, 2, 3	–	I, II	–	Ax	–	T1D, O	KPC-2	CTX-M-8
1 897 570	ST2655-1LV	2011	Other	F	KL7	O2v2	23/10	2, 4	–	I, II	–	–	K36	T1D, O+, S	GES-5	CTX-M-8
1 412 708	ST340	2010	Tissue	A	KL15	O4	18/9	1, 2, 3, 4	1, 2, 3	I, II	D	A12	–	L2, T1D	KPC-2	CTX-M-15,8^
1 414 704	ST340	2010	Tissue	A	KL15	O4	19/9	1, 2, 3, 4	1, 2	I, II	D	A12	–	T1D	KPC-2	CTX-M-15
1 450 421	ST340	2010	Blood	A	KL15	O4	23/11	2	1, 2, 3	I, II	D	Ax	–	L2, T1D	KPC-2	CTX-M-15
1 465 945	ST340	2010	Urinary	A	KL15	O4	22/11	1, 2, 3	1, 2	I,II	D	A12	–	T1D	KPC-2	CTX-M-15
1 877 899	ST340	2011	Other	A	KL151	O4	22/11	2	1, 2	–	D	Ax	–	–	–	CTX-M-15
2 210 477	ST340	2012	Blood	A	KL64	O4	22/11	1, 2, 3	1, 2, 3, 5	I, II	AD	A1, A12+	–	L2, O2, T1D, O^+^	KPC-2	CTX-M-15,2
2 703 880	ST340	2012	Pulm.	A	KL15	O4	22/9	1, 2, 3, 4	1, 2		–	A12+	–	T1D	KPC-2	CTX-M-15
3 049 866	ST340	2012	Pulm.	G	KL15	O4	22/11	1, 2, 3, 4	1, 2		–	A12+	–	T1D	KPC-2	CTX-M-15
3 049 874	ST340	2012	Urinary	G	KL15	O4	22/11	1, 2, 3, 4	1, 2		–	A12+	–	T1D	KPC-2	CTX-M-15
3 297 576	ST340	2012	Blood	G	KL15	O4	20/9	1, 2, 3, 4	1, 2		–	A12	–	T1D	KPC-2	CTX-M-15
**4 022 578**	ST340	2013	Pulm.	D	KL151	O4††	22/10	2, 3	1, 2	I, II	–	A12	K36	O2	KPC-2	CTX-M-15
4 152 813	ST340	2013	Urinary	G	KL151††	O4	21/10	2, 3	1, 2	I, II	–	A12	–	O2	KPC-2	CTX-M-15
4 281 075	ST340	2013	Wound	G	KL15	O4	21/10	3, 4	1, 2, 5		–	A12+	–	–	KPC-2	–
**4 879 999**	ST340	2013	Pulm.	H	KL151	O4	18/9	2, 3, 4	1, 2		–	A12+	K36	O	KPC-2	CTX-M-8
6 264 345	ST340	2014	Pulm.	D	KL15	O4	19/9	1, 2	1, 2	–	–	A14	–	T1D	KPC-2	CTX-M-15
6 334 725	ST340	2014	Urinary	A	KL151	O4	19/9	2, 3	1, 2	I, II	–	A12+	–	–	NDM-1	CTX-M-8
4 455 550	ST37	2013	Urinary	H	KL55	OL103	23/9	2, 3	1, 2	II	–	A12+	–	–	NDM-1	–
4 552 909	ST37	2013	Urinary	H	KL55	OL103	23/11	2, 3	1, 2, 5	I, II	D	A12+	–	–	NDM-1	CTX-M-27
4 941 726	ST37	2013	Urinary	D	KL55	OL103	18/9	2, 3	1, 2, 5	I, II	–	A12+	–	–	NDM-1	–
5 240 651	ST37	2013	Urinary	E	KL55	OL103	19/9	2, 3	1, 2, 5	I, II	D	A12+	–	–	NDM-1	CTX-M-27
6 533 645	ST37	2014	Rectal	K	KL55	OL103	19/10	2, 3	1, 2, 5	II	D	A12+	–	–	NDM-1	CTX-M-27
5 700 769	ST437	2014	Blood	G	KL36	O4	20/9	2, 4	1, 2, 5		AD	Axe‡‡	–	T1D, O+	KPC-2	CTX-M-15

*Bold text indicates that these strains encode yersiniabactin on the *ybt9* of the integrative and conjugative element ICE*Kp*3.

†AGMs code 1, AphA6; 2, any Aac; 3, any Aad; 4, other Aph ignored StrAB SatA Rmt.

‡FQ, fluoroquinolone-resistance mutations; code 1, GyrA-83I; 2, ParC-80I; 3, Qnr-S1; 4, other GyrA; 5, other Qnr.

§Sulfonamide-resistance: I, SulI; II, SulII.

||Tetracycline-resistance efflux: A, TetA; D, TetD.

¶Trimethoprim.

#Porin mutations that might affect antimicrobial uptake and susceptibility.

**L2, LAP-2; O2, Oxa-2; T1D, TEM-1D^; O+, OXA-2 or −9; S, SHV-28 or −187.

††Missing genes.

‡‡For each potential resistance gene identified by Kleborate, this indicates inexact nucleotide and inexact amino acid match; with ^ indicating inexact nucleotide but exact amino acid match; ? for incomplete match.

## Supplementary Data

Supplementary material 1

Supplementary material 2

## References

[1] Azevedo PAA, Furlan JPR, Goncalves GB, Gomes CN, Goulart RDS (2019). Molecular characterisation of multidrug-resistant *Klebsiella pneumoniae* belonging to CC258 isolated from outpatients with urinary tract infection in Brazil. J Glob Antimicrob Resist.

[2] Zhou K, Xiao T, David S, Wang Q, Zhou Y (2020). Novel subclone of carbapenem-resistant *Klebsiella pneumoniae* sequence type 11 with enhanced virulence and transmissibility, China. Emerg Infect Dis.

[3] Martin WJ, Yu PK, Washington JA (1971). Epidemiologic significance of *Klebsiella pneumoniae:* a 3-month study. Mayo Clin Proc.

[4] Daikos GL, Markogiannakis A, Souli M, Tzouvelekis LS (2012). Bloodstream infections caused by carbapenemase-producing *Klebsiella pneumoniae*: a clinical perspective. Expert Rev Anti Infect Ther.

[5] Paczosa MK, Mecsas J (2016). *Klebsiella pneumoniae*: going on the offense with a strong defense. Microbiol Mol Biol Rev.

[6] Bi W, Liu H, Dunstan RA, Li B, Torres VVL (2017). Extensively drug-resistant *Klebsiella pneumoniae* causing nosocomial bloodstream infections in China: molecular investigation of antibiotic resistance determinants, informing therapy, and clinical outcomes. Front Microbiol.

[7] Haller S, Kramer R, Becker K, Bohnert JA, Eckmanns T (2019). Extensively drug-resistant *Klebsiella pneumoniae* ST307 outbreak, north-eastern Germany, June to October 2019. Euro Surveill.

[8] Huang Y-H, Chou S-H, Liang S-W, Ni C-E, Lin Y-T (2018). Emergence of an XDR and carbapenemase-producing hypervirulent *Klebsiella pneumoniae* strain in Taiwan. J Antimicrob Chemother.

[9] Andrade LN, Vitali L, Gaspar GG, Bellissimo-Rodrigues F, Martinez R (2014). Expansion and evolution of a virulent, extensively drug-resistant (polymyxin B-resistant), QnrS1-, CTX-M-2-, and KPC-2-producing *Klebsiella pneumoniae* ST11 international high-risk clone. J Clin Microbiol.

[10] Martin RM, Bachman MA (2018). Colonization, infection, and the accessory genome of *Klebsiella pneumoniae*. Front Cell Infect Microbiol.

[11] Centers for Disease Control and Prevention (2013). Vital signs: carbapenem-resistant *Enterobacteriaceae*. Morb Mortal Wkly Rep.

[12] Karaiskos I, Giamarellou H (2014). Multidrug-resistant and extensively drug-resistant Gram-negative pathogens: current and emerging therapeutic approaches. Expert Opin Pharmacother.

[13] Wyres KL, Wick RR, Judd LM, Froumine R, Tokolyi A (2019). Distinct evolutionary dynamics of horizontal gene transfer in drug resistant and virulent clones of *Klebsiella pneumoniae*. PLoS Genet.

[14] Petersen FC, Scheie AA (2000). Genetic transformation in *Streptococcus mutans* requires a peptide secretion-like apparatus. Oral Microbiol Immunol.

[15] Holt KE, Wertheim H, Zadoks RN, Baker S, Whitehouse CA (2015). Genomic analysis of diversity, population structure, virulence, and antimicrobial resistance in *Klebsiella pneumoniae*, an urgent threat to public health. Proc Natl Acad Sci USA.

[16] Bolger AM, Lohse M, Usadel B (2014). Trimmomatic: a flexible trimmer for Illumina sequence data. Bioinformatics.

[17] Magoc T, Salzberg SL (2011). FLASH: fast length adjustment of short reads to improve genome assemblies. Bioinformatics.

[18] Bankevich A, Nurk S, Antipov D, Gurevich AA, Dvorkin M (2012). SPAdes: a new genome assembly algorithm and its applications to single-cell sequencing. J Comput Biol.

[19] Shen W, Le S, Li Y, Hu F (2016). SEQKIT: a cross-platform and ultrafast toolkit for FASTA/Q file manipulation. PLoS One.

[20] Gurevich A, Saveliev V, Vyahhi N, Tesler G (2013). QUAST: quality assessment tool for genome assemblies. Bioinformatics.

[21] Seemann T (2014). Prokka: rapid prokaryotic genome annotation. Bioinformatics.

[22] Inouye M, Dashnow H, Raven LA, Schultz MB, Pope BJ (2014). SRST2: Rapid genomic surveillance for public health and hospital microbiology labs. Genome Med.

[23] Wyres KL, Wick RR, Gorrie C, Jenney A, Follador R (2016). Identification of *Klebsiella* capsule synthesis loci from whole genome data. Microb Genom.

[24] Lam MMC, Wick RR, Wyres KL, Gorrie CL, Judd LM (2018). Genetic diversity, mobilisation and spread of the yersiniabactin-encoding mobile element ICEKp in *Klebsiella pneumoniae* populations. Microb Genom.

[25] Lam MMC, Wyres KL, Judd LM, Wick RR, Jenney A (2018). Tracking key virulence loci encoding aerobactin and salmochelin siderophore synthesis in *Klebsiella pneumoniae*. Genome Med.

[26] Wick RR, Heinz E, Holt KE, Wyres KL (2018). Kaptive web: user-friendly capsule and lipopolysaccharide serotype prediction for *Klebsiella* genomes. J Clin Microbiol.

[27] Sheppard AE, Stoesser N, German-Mesner I, Vegesana K, Walker AS (2018). TETyper: a bioinformatic pipeline for classifying variation and genetic contexts of transposable elements from short-read whole-genome sequencing data. Microb Genom.

[28] Page AJ, Cummins CA, Hunt M, Wong VK, Reuter S (2015). Roary: rapid large-scale prokaryote pan genome analysis. Bioinformatics.

[29] Page AJ, Taylor B, Delaney AJ, Soares J, Seemann T (2016). SNP-sites: rapid efficient extraction of SNPs from multi-FASTA alignments. Microb Genom.

[30] Stamatakis A (2014). RAxML version 8: a tool for phylogenetic analysis and post-analysis of large phylogenies. Bioinformatics.

[31] Jolley KA, Maiden MC (2010). BIGSdb: scalable analysis of bacterial genome variation at the population level. BMC Bioinformatics.

[32] Kans J (2021). Entrez Direct: E-utilities on the Unix Command Line. Entrez Programming Utilities Help.

[33] Madeira F, Park YM, Lee J, Buso N, Gur T (2019). The EMBL-EBI search and sequence analysis tools APIs in 2019. Nucleic Acids Res.

[34] Bialek-Davenet S, Criscuolo A, Ailloud F, Passet V, Jones L (2014). Genomic definition of hypervirulent and multidrug-resistant *Klebsiella pneumoniae* clonal groups. Emerg Infect Dis.

[35] Cortes G, Borrell N, de Astorza B, Gomez C, Sauleda J (2002). Molecular analysis of the contribution of the capsular polysaccharide and the lipopolysaccharide O side chain to the virulence of *Klebsiella pneumoniae* in a murine model of pneumonia. Infect Immun.

[36] Gupta SK, Padmanabhan BR, Diene SM, Lopez-Rojas R, Kempf M (2014). ARG-ANNOT, a new bioinformatic tool to discover antibiotic resistance genes in bacterial genomes. Antimicrob Agents Chemother.

[37] Cannatelli A, Giani T, D’Andrea MM, Pilato D, Arena F (2014). MgrB inactivation is a common mechanism of colistin resistance in KPC-producing *Klebsiella pneumoniae* of clinical origin. Antimicrob Agents Chemother.

[38] Jayol A, Poirel L, Brink A, Villegas MV, Yilmaz M (2014). Resistance to colistin associated with a single amino acid change in protein PmrB among *Klebsiella pneumoniae* isolates of worldwide origin. Antimicrob Agents Chemother.

[39] Sugawara E, Kojima S, Nikaido H (2016). *Klebsiella pneumoniae* major porins OmpK35 and OmpK36 allow more efficient diffusion of beta-lactams than their *Escherichia coli* homologs OmpF and OmpC. J Bacteriol.

[40] Fajardo-Lubian A, Ben Zakour NL, Agyekum A, Qi Q, Iredell JR (2019). Host adaptation and convergent evolution increases antibiotic resistance without loss of virulence in a major human pathogen. PLoS Pathog.

[41] Kumar MRR, Arunagirinathan N, Srivani S, Dhanasezhian A, Vijaykanth N (2017). Dissemination of trimethoprim-sulfamethoxazole drug resistance genes associated with class 1 and class 2 integrons among gram-negative bacteria from HIV patients in South India. Microb Drug Resist.

[42] Aldred KJ, Kerns RJ, Osheroff N (2014). Mechanism of quinolone action and resistance. Biochemistry.

[43] Nordmann P, Poirel L, Walsh TR, Livermore DM (2011). The emerging NDM carbapenemases. Trends Microbiol.

[44] Carvalho-Assef AP, Pereira PS, Albano RM, Beriao GC, Chagas TP (2013). Isolation of NDM-producing *Providencia rettgeri* in Brazil. J Antimicrob Chemother.

[45] Rozales FP, Ribeiro VB, Magagnin CM, Pagano M, Lutz L (2014). Emergence of NDM-1-producing *Enterobacteriaceae* in Porto Alegre. Int J Infect Dis.

[46] Carneiro M, Goncalves RA, de Souza JG, Teixeira CB, Krummenauer EC (2014). New carbapenases in Brazil. Expert Rev Anti Infect Ther.

[47] Stoesser N, Giess A, Batty EM, Sheppard AE, Walker AS (2014). Genome sequencing of an extended series of NDM-producing *Klebsiella pneumoniae* isolates from neonatal infections in a Nepali hospital characterizes the extent of community- versus hospital-associated transmission in an endemic setting. Antimicrob Agents Chemother.

[48] Poirel L, Al Maskari Z, Al Rashdi F, Bernabeu S, Nordmann P (2011). NDM-1-producing *Klebsiella pneumoniae* isolated in the Sultanate of Oman. J Antimicrob Chemother.

[49] Williamson DA, Sidjabat HE, Freeman JT, Roberts SA, Silvey A (2012). Identification and molecular characterisation of New Delhi metallo-beta-lactamase-1 (NDM-1)- and NDM-6-producing *Enterobacteriaceae* from New Zealand hospitals. Int J Antimicrob Agents.

[50] Giske CG, Froding I, Hasan CM, Turlej-Rogacka A, Toleman M (2012). Diverse sequence types of *Klebsiella pneumoniae* contribute to the dissemination of blaNDM-1 in India, Sweden, and the United Kingdom. Antimicrob Agents Chemother.

[51] Magiorakos AP, Srinivasan A, Carey RB, Carmeli Y, Falagas ME (2012). Multidrug-resistant, extensively drug-resistant and pandrug-resistant bacteria: an international expert proposal for interim standard definitions for acquired resistance. Clin Microbiol Infect.

[52] Pitout JDD, Nordmann P, Poirel L (2015). Carbapenemase-producing *Klebsiella pneumoniae*, a key pathogen set for global nosocomial dominance. Antimicrob Agents Chemother.

[53] Palmeiro JK, de Souza RF, Schörner MA, Passarelli-Araujo H, Grazziotin AL (2019). Molecular epidemiology of multidrug-resistant *Klebsiella pneumoniae* isolates in a Brazilian tertiary hospital. Front Microbiol.

[54] Lee JH, Lee JH, Park KS, Kim YB, Jeong BC (2016). Global dissemination of carbapenemase-producing *Klebsiella pneumoniae*: epidemiology, genetic context. Front Microbiol.

[55] Pereira PS, de Araujo CF, Seki LM, Zahner V, Carvalho-Assef AP (2013). Update of the molecular epidemiology of KPC-2-producing *Klebsiella pneumoniae* in Brazil: spread of clonal complex 11 (ST11, ST437 and ST340. J Antimicrob Chemother.

[56] Andrade LN, Curiao T, Ferreira JC, Longo JM, Climaco EC (2011). Dissemination of blaKPC-2 by the spread of *Klebsiella pneumoniae* clonal complex 258 clones (ST258, ST11, ST437) and plasmids (IncFII, IncN, IncL/M) among *Enterobacteriaceae* species in Brazil. Antimicrob Agents Chemother.

[57] Bartolleti F, Seco BM, Capuzzo Dos Santos C, Felipe CB, Lemo ME (2016). Polymyxin B resistance in carbapenem-resistant *Klebsiella pneumoniae*, Sao Paulo, Brazil. Emerg Infect Dis.

