# Supplementary material for: Genomic insights into the diversity, virulence and resistance of Klebsiella pneumoniae extensively drug resistant clinical isolates
Source: Microb Genom. 2021 Aug 23;7(8):000613. doi: 10.1099/mgen.0.000613 (PMC8549359; doi:10.1099/mgen.0.000613)
Supplement: Supplementary material 1 [file mgen-7-0613-s001.pdf]

SUPPLEMENTAL FIGURES

A.

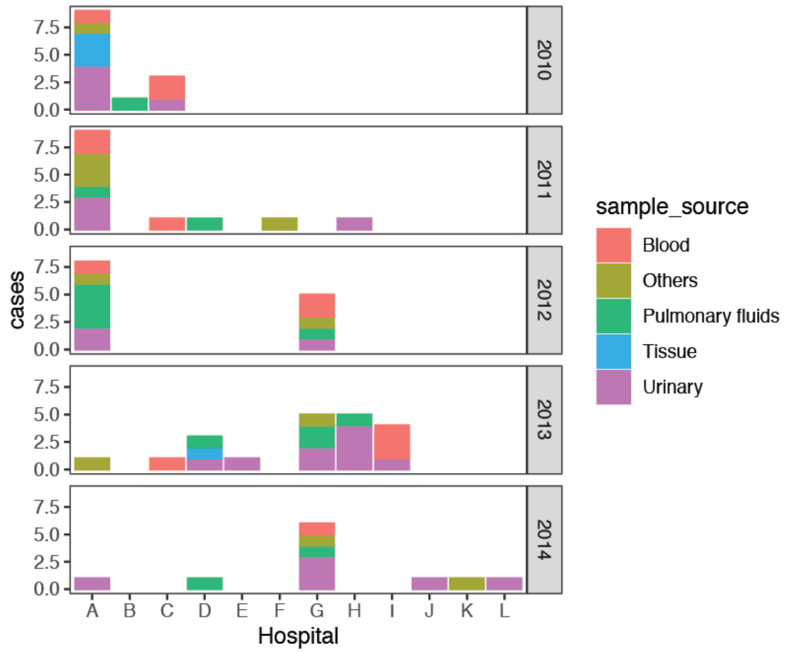

B.

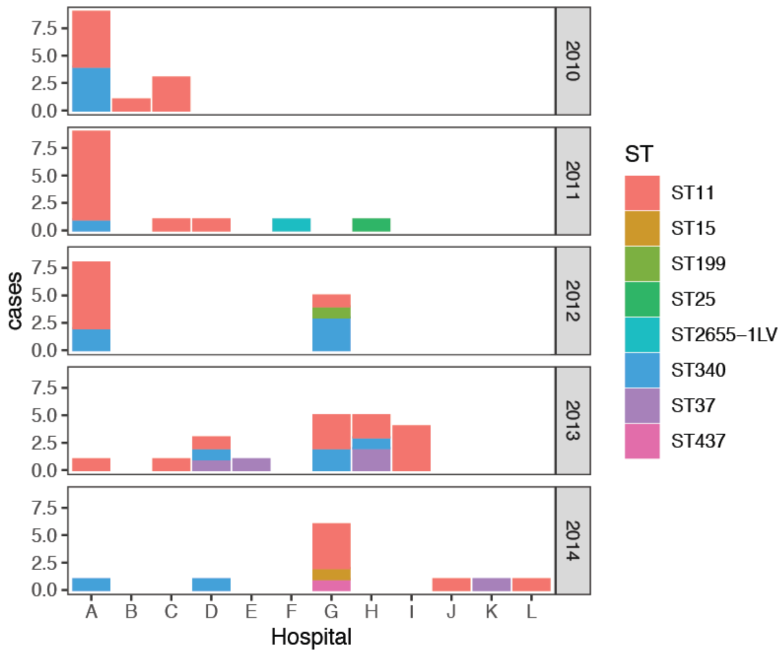

Supplemental Figure S1

Supplemental Figure S1. *K. pneumoniae* clinical isolates from twelve Brasília's hospitals between 2010-2014 with the number of cases per isolation sites (**A**) and sequence types, ST (**B**) plotted per year.

A.

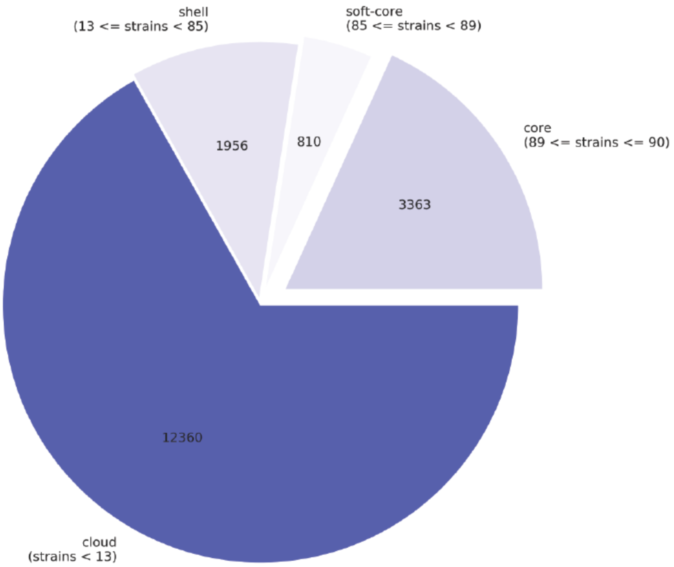

B.

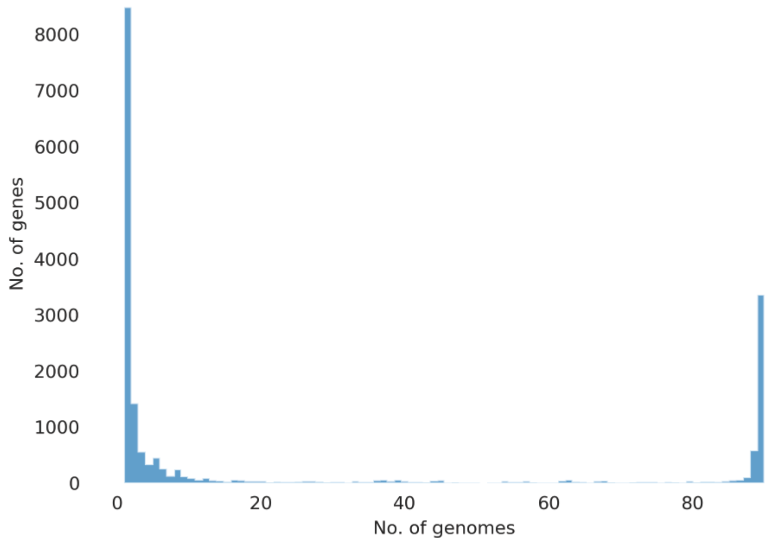

Supplemental Figure S2

Supplemental Figure S2. R**oary pan-genome analysis of the 70 Brasília isolates and the 20 reference *K. pneumoniae* genomes**: (**A**) Identification of 3,363 genes present in >99% of strains, 810 genes present in ≥95% and <99% of strains, 1956 genes present in ≥15% and <95% and 12,360 genes present in <15% of strains. (**B**) The number of genes present as a function of the number of genomes. The peak at 1 genome shows that many of the genes that were different between strains were unique.

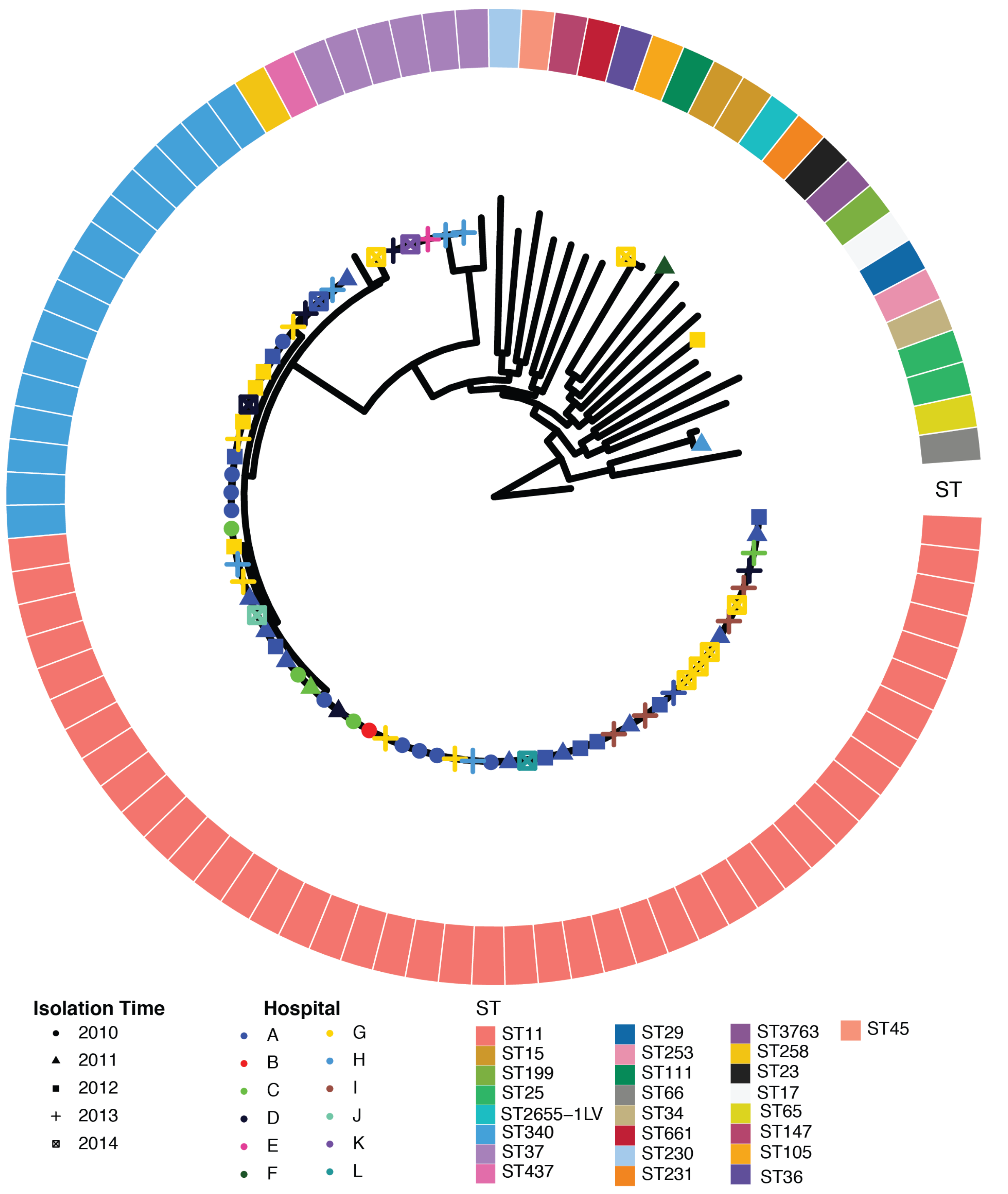

Supplemental Figure S3

Supplemental Figure S3. ST groups of *K. pneumoniae* clinical isolates from twelve Brasília's hospitals. *K. pneumoniae* clinical isolates sampled between 2010-2014 with time (shown by different shapes for the tip labels), hospital (in different colors for the tip labels) and ST (color ring) mapped onto a circular core-genome SNP phylogeny.

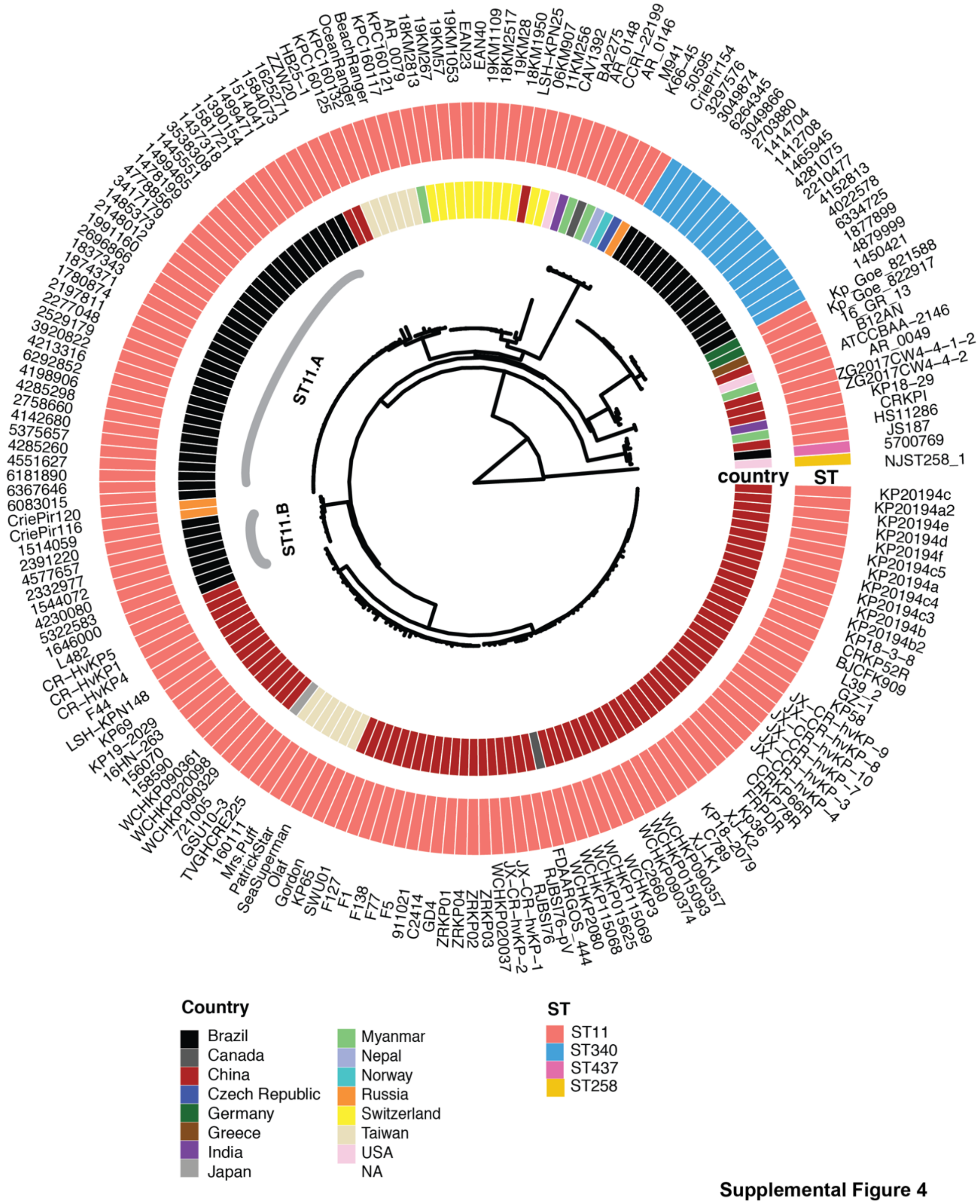

Supplemental Figure 4

Supplemental Figure S4. Global distribution of ST11 strains and Brasília *K. pneumoniae* CC258 clinical isolates. Core-genome SNP phylogeny was constructed using CC258 Brasília isolates (ST11, ST340, ST437), 128 reference-quality genomes of ST11 strains from around the world, with reference-quality NJST258_1 genome as the outgroup. The inner color ring indicating country of isolation, with the outer color ring indicating ST groups.

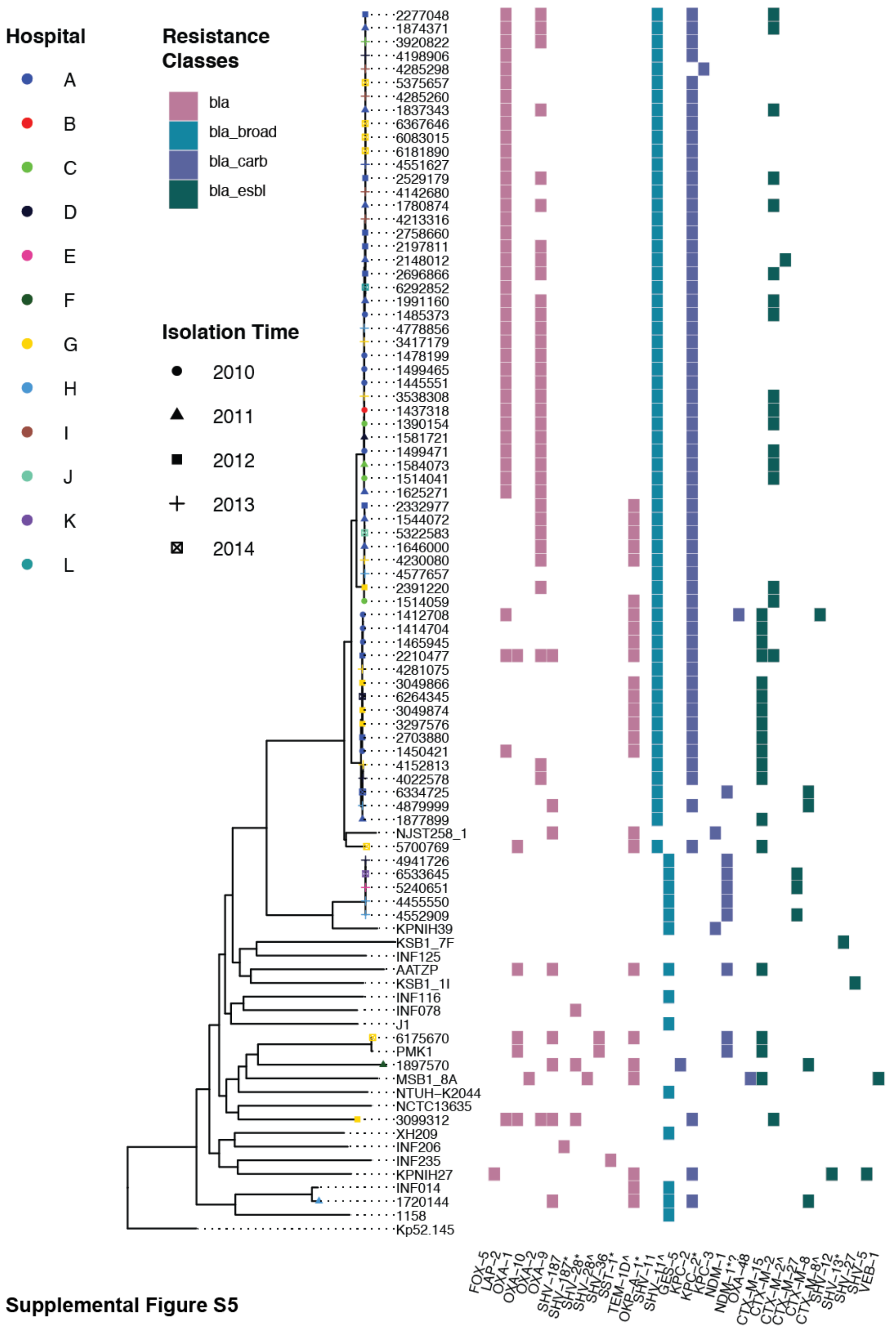

Supplemental Figure S5

Supplemental Figure S5. Beta-lactamase resistance of *K. pneumoniae* clinical isolates from twelve Brasília's hospitals. Isolates sampled between 2010-2014 with time (shown by different shapes annotating the phylogenetic tree tips) and hospital (shown by different colors annotating the phylogenetic tree tips), as well as the 20 reference genomes, mapped onto a core-genome SNP phylogeny. The presence of different resistance genes is colored by classes: bla (β-lactamases), bla_broad (broad spectrum β-lactamases), bla_carb (carbapenemases) and bla_esbl (extended spectrum β-lactamases).

SUPPLEMENTAL TABLE LEGENDS

Table S1. Metadata for 70 sequenced *K. pneumoniae* strains, including sampling year, source, hospital, isolate ID (LACEN ID), and sequencing ID.

Table S2. Sequencing depth for 70 *K. pneumoniae* strains

Table S3. Twenty-five Brasília *Klebsiella* isolates excluded in this study, unique ENA Run identifier, the associated LACEN ID (where available), sequencing depth and exclusion criteria.

Table S4. Twenty reference *K. pneumoniae* genomes from diverse clonal groups and their respective GenBank Accession Number included in this study.

Table S5. Reference *K. pneumoniae* genomes (n=677) from diverse clonal groups and their respective ST group.

Table S6. Kleborate analyses results for 70 *K. pneumoniae* strains from this study and the 20 reference genomes.

Table S7. Quast assembly quality control for 70 sequenced *K. pneumoniae* strains

Table S8. Roary pan-genome analyses summary statistics.

Table S9. Vitek2 antibiotic resistance profile for 70 sequenced *K. pneumoniae* strains, with yellow highlighting resistance based on EUCAST clinical breakpoint.
